# Pentoxifylline Prevents Autoimmune Mediated Inflammation in Low Dose Streptozotocin Induced Diabetes

**DOI:** 10.1155/2001/37209

**Published:** 2001

**Authors:** Stanislava D. Stošić-Grujičić, Danijela D. Maksimović, Marija B. Mostarica Stojković, Miodrag L. Lukić

**Affiliations:** 1 lnstitute for Biological Research “Siniša Stanković” 29. Novembra 142 Belgrade 11000 Serbia and Montenegro; 2 lnstitute of Microbiology and Immunology School of Medicine Belgrade University Dr Subotića 1 Belgrade 11000 Serbia and Montenegro; 3 Immunology Unit Department of Medical Microbiology Faculty of Medicine & Health Sciences United Arab Emirates University Al Ain United Arab Emirates

**Keywords:** Autoimmune Diabetes,, Insulitis, Interferon-gamma, Nitric Oxide, Pentoxifylline

## Abstract

Xanthine derivative, pentoxifylline (PTX), has been recently shown to exert a protective effects in certain animal models of autoimmunity, including diabetes in NOD mice. In the present study, the immunomodulatory potential of PTX was investigated in autoimmune diabetes induced by multiple low doses of streptozotocin (MLD-SZ) in genetically susceptible CBA/H mice (tested with 40 mg SZ/kg b.w. for 5 days) and DA rats (tested with 20 mg/kg b.w. for 5 days). In both species, 2 – 3 weeks following the MLD-SZ treatment, sustained hyperglycemia developed, as an outcome of inflammatory reaction with endothelial cell activation and accumulation of mononuclear cells. Although there was no evidence of typical insulitis in early disease development (day 10), in both rats and mice, macrophages, CD4^+^ and CD8^+^ cells were present in the islets of Langerhans as diffuse mononuclear infiltrates with the expression of IFN-γ and inducible NO synthase (iNOS). Administration of PTX (200 mg/kg/day for 10 days) in combination with MLD-SZ reduced insulitis and the production of mediators tested, and prevented the development of hyperglycemia. These results suggest that beneficial effects of PTX involve down-regulation of local proinflammatory cytokine-mediated NO synthase pathway. They also demonstrate that in addition to ameliorating spontaneous autoimmunity in NOD mice, PTX may be effective in downregulating an inflammatory autoimmune process triggered in susceptible host by an external agents, such as streptozotocin.


*Developmental Immunology*, 2001, Vol. 8(3-4), pp. 213-221
Reprints available directly from the publisher
Photocopying permitted by license only

© 2001 OPA (Overseas Publishers Association)
N.V. Published by license under
the Harwood Academic Publishers imprint,
part of The Gordon and Breach Publishing Group,
a member of the Taylor and Francis Group

### Pentoxifylline Prevents Autoimmune Mediated Inflammation in Low Dose Streptozotocin Induced Diabetes

STANISLAVA D. STOŠIĆ-GRUJIČIĆ[a]*, DANIJELA D. MAKSIMOVIĆ[a], MARIJA B. MOSTARICA STOJKOVIĆ[b] and MIODRAG L. LUKIĆ[b†]

[a]*Institute for Biological Research "Siniša Stanković", 29. Novembra 142, 11000 Belgrade Yugoslavia and* [b]*Institute of Microbiology and Immunology, School of Medicine, Belgrade University, Dr Subotića 1, 11000 Belgrade, Yugoslavia*

Xanthine derivative, pentoxifylline (PTX), has been recently shown to exert a protective effects in certain animal models of autoimmunity, including diabetes in NOD mice. In the present study, the immunomodulatory potential of PTX was investigated in autoimmune diabetes induced by multiple low doses of streptozotocin (MLD-SZ) in genetically susceptible CBA/H mice (tested with 40 mg SZ/kg b.w. for 5 days) and DA rats (tested with 20 mg/kg b.w. for 5 days). In both species, 2 – 3 weeks following the MLD-SZ treatment, sustained hyperglycemia developed, as an outcome of inflammatory reaction with endothelial cell activation and accumulation of mononuclear cells. Although there was no evidence of typical insulitis in early disease development (day 10), in both rats and mice, macrophages, $CD4^+$ and $CD8^+$ cells were present in the islets of Langerhans as diffuse mononuclear infiltrates with the expression of IFN-$\gamma$, and inducible NO synthase (iNOS). Administration of PTX (200 mg/kg/day for 10 days) in combination with MLD-SZ reduced insulitis and the production of mediators tested, and prevented the development of hyperglycemia. These results suggest that beneficial effects of PTX involve down-regulation of local proinflammatory cytokine-mediated NO synthase pathway. They also demonstrate that in addition to ameliorating spontaneous autoimmunity in NOD mice, PTX may be effective in downregulating an inflammatory autoimmune process triggered in susceptible host by an external agents, such as streptozotocin.

*Keywords:* Autoimmune Diabetes, Insulitis, Interferon-gamma, Nitric Oxide, Pentoxifylline

### INTRODUCTION

Pentoxifylline (PTX) [3,7-dimethyl-1-(5-oxohexyl) xanthine] is the drug that is widely used for the treatment of vascular disorders. However, there are new evidences for the potential therapeutic properties of this compound. Preclinical studies in laboratory rodents have shown that PTX and related compounds may favorably influence the course of experimental inflammatory and autoimmune disorders, such as autoimmune encephalomyelitis (Rott et al., 1993; Nataf et al., 1993), meningitis (Saez-Lorens et al., 1990) and autoimmune neuritis (Constantinescu et al., 1996).

* Correspondence: Dr. Stanislava Stošić-Grujičić Institute for Biological Research "Siniša Stanković" 29. Novembra 142 11000 Belgrade Yugoslavia Tel/fax: +38111–657–258; E-mail: DUTA@eunet.yu
† Present address: Immunology Unit, Department of Medical Microbiology, Faculty of Medicine & Health Sciences, United Arab Emirates University, Al Ain, U.A.E.

Insulin-dependent diabetes mellitus is characterized by a failure of self-tolerance leading to Thl cell – mediated autoimmune attack leading to destruction of insulin-producing β cells located in the islets of Langerhans. While high doses of β-cell toxin streptozotocin (SZ) induce diabetes through a direct toxic effect on β-cells, multiple low doses in susceptible strains of mice and rats initiate an autoimmune destructive process similar to that observed in human disease (Like and Rossini, 1976; Lukić et al., 1991a; Kolb and Kroncke, 1993). Therefore, MLD-SZ induced diabetes allows for the study on agents potentially designed to modulate β-cell specific autodestructive process. However, it is not known whether there are any differences or similarities between spontaneously and experimentally induced diabetic animal models in the mode of action of different immunomodulatory drugs. Recently Liang et al (1998) have demonstrated that PTX inhibits secretion of IL-12 by macrophages and IFN-γ by Th1 cells. It appeared that this effect may be responsible for the amelioration of insulitis in NOD mice (Liang et al., 1998). Our data therefore strengthen the notion that similar effector mechanisms are operative in spontaneous and toxin induced autoimmune diabetes. By using the model of diabetes induced by MLD-SZ we demonstrated that PTX, similarly to spontaneous models of diabetes (Liang et al., 1998), suppresses development of hyperglycemia mainly by downregulating the development of inflammatory lesions in the pancreata.

### RESULTS

### Effects of PTX on Hyperglycemia

In order to evaluate the ability of PTX to interfere with diabetogenic process induced by an external agents, streptozotocin, we used both murine and rat model of autoimmune diabetes induction. Although the dose regimens of β-cell toxin SZ for mice and rats were not identical, in both rodent species, as already reported (Lukić et al., 1991a; Lukić et al., 1991b), repeated injections with 5 subtoxic daily doses of SZ induced delayed hyperglycemia 10 to 20 days after completion of the treatment (Figure 1). *In vivo* treatment of animals with 10 consecutive constant doses (200 mg/kg/day) of PTX significantly reduces hyperglycemia in both experimental species. The protective effect of the drug is long-lasting, at least for 8 weeks after the end of the treatment when experiment was terminated (Figure 1).

### Cellular Changes

The interference of PTX with a pathological process leading to islet dysfunction have further been studied by immunological and immunohistochemical analysis at the level of target tissue. Histological analysis and comparison with untreated control was done by day 10 after the induction of the disease and completion of the PTX treatment, and by day 56 of the monitoring of hyperglycemia. In comparison to normal architecture of the islets found in animals without any treatment, on day 10 in control MLD-SZ-induced animals histopathological changes are evident. Analysis in some of the islets of susceptible CBA/H mice at this time revealed heavy mononuclear infiltrates (Figure 2). In susceptible DA rats small infiltrates were rarely seen (grade 1) and infiltrating cells were scattered throughout the islets (Figure 3). In both species mononuclear infiltration was accompanied with initial necrotic changes and slight edema in the connective spaces around the islets and blood vessels (Figures 2 and 3). However, the majority of the islets were still well preserved and free from infiltration, thus suggesting asynchronous process. Immunohistochemical analysis in rats confirmed our earlier findings (Lukić et al., 1991a) that mononuclear cells participating in the insulitis process were $CD4^+$ and $CD8^+$ lymphocytes, as well as blood borne $ED1^+$ macrophages. Some mononuclear cells within the islets also were positively stained for MHC ClassII. At the later stage of the disease (day 56) most of the islets lost clear margins and their characteristic structure. In both species progression of insulitis led to more severe necrotic changes throughout the affected islets (grade 2 prevailed), such as vacuolization related to focal necrosis and picnosis of the islet cells (data not shown). Treatment with PTX concomi-

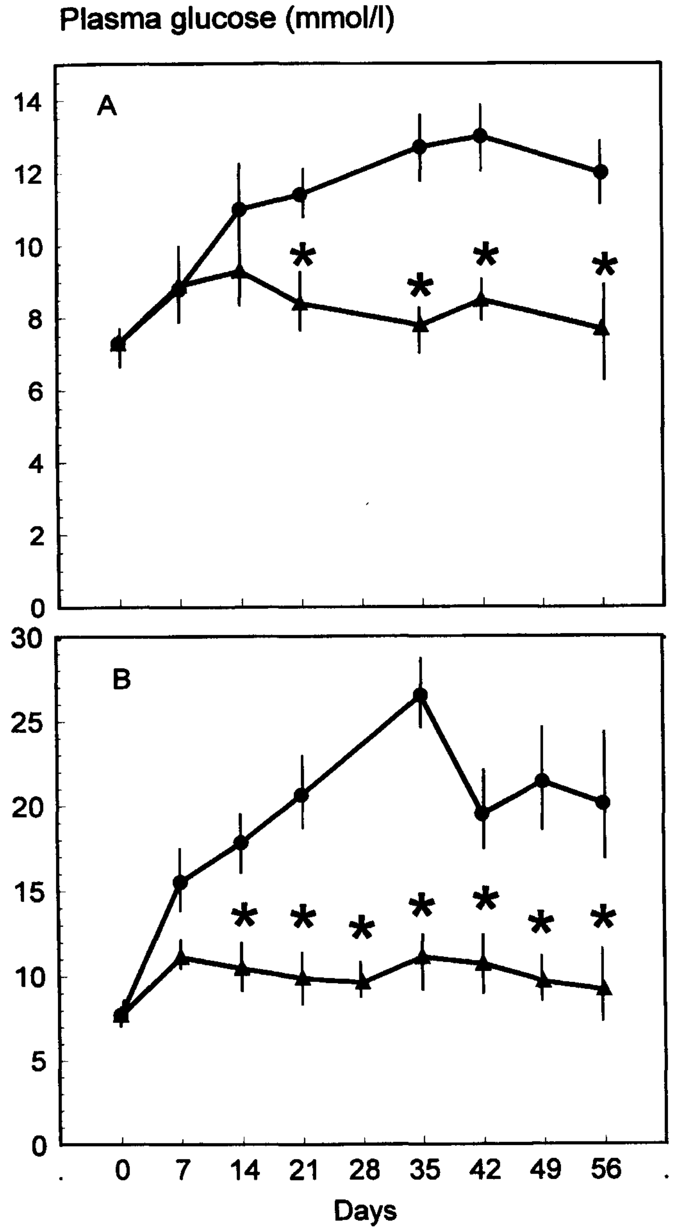

FIGURE 1 Effects of PTX treatment on the development of MLD-SZ-induced hyperglycemia in mice and rats. Plasma glucose levels, determined in CBA/H mice (A) and DA rats (B) receiving MLD-SZ for 5 consecutive days (•), or animals treated with MLD-SZ in conjunction with PTX from day 0 to day 9 in relation to the first SZ injection (▲). Significantly different from the value of MLD-SZ-treated but non-PTX-treated control animals: *$P<0.05$; Student's t-test tantly with MLD-SZ significantly ameliorated histopathological changes and down-regulated influx of inflammatory cells. In comparison with control MLD-SZ-treated animals, on day 10 in both species there were much larger numbers of normal islets (grade 0), or islets with only mild mononuclear infil-

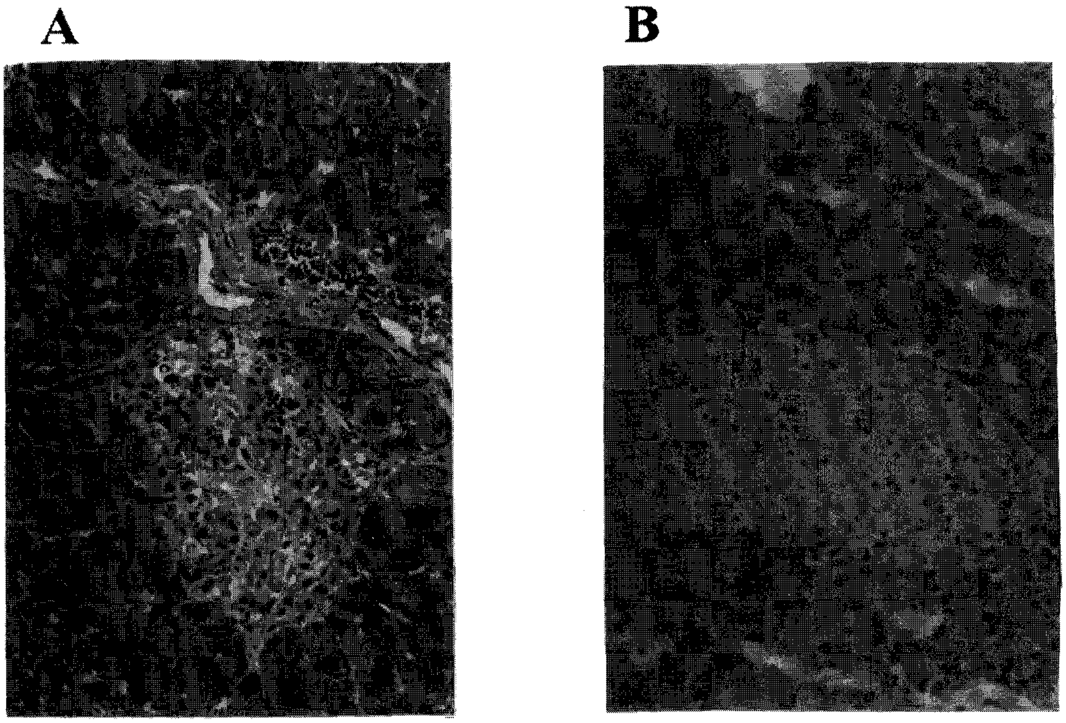

FIGURE 2 Effect of PTX treatment on the development of MLD-SZ-induced insulitis in CBA/H mice. (A) Histology of pancreatic islet by day 10 after MLD-SZ treatment (HE × 60). Note massive infiltration of the islet with the initial necrotic changes and endothelial swelling. (B) Histology of pancreatic islet by day 10 after treatment with MLD-SZ + PTX (HE × 50). Note well preserved islets without insulitis and necrosis and with normal endothel trates (Figures 2 and 3). At this time, inflammatory cells with the characteristic phenotype were virtually absent, or rare and spread only to the periphery of the islets (Figure 3). Vascular dilatation with hypertrophy of the endothelium was hardly observed. In the later period, in comparison to PTX nontreated diabetic animals, hypocellularity and atrophy of the islets were less prominent and most of the islets still remained intact. In general, although PTX prophylaxis could not completely suppress insulitis development and β-cell damage, it drastically reduced it severity, which was sufficient for the normoglycemic status of the animals (Figure 1).

### Molecular Changes

To investigate the effects of PTX on the molecular alterations accompanying islet destruction, we analyzed immunohystochemically the presence of proinflammatory mediators (IFN-γ and iNOS) – in the islets. In control MLD-SZ-induced diabetic animals mononuclear cell infiltration in pancreata was accompanied by prominent expression of IFN-γ and iNOS (Figure 4). In addition to intraislet cells, marked iNOS expression was observed on the endothelial cells of the hypertrophic blood vessels. By contrast, in the PTX-treated group, in some of the islets, rare IFN-$\gamma^+$ and iNOS$^+$ cells were evenly dispersed throughout, but most of the remaining islets were completely negative (Figure 4), similarly to healthy nontreated animals. Concordantly with diabetic status, in MLD-SZ-treated animals impaired insulin secretion was found, as revealed by insulin staining of pancreata, while treatment with PTX resulted in unaltered distribution of insulin positive β-cells (Figure 4).

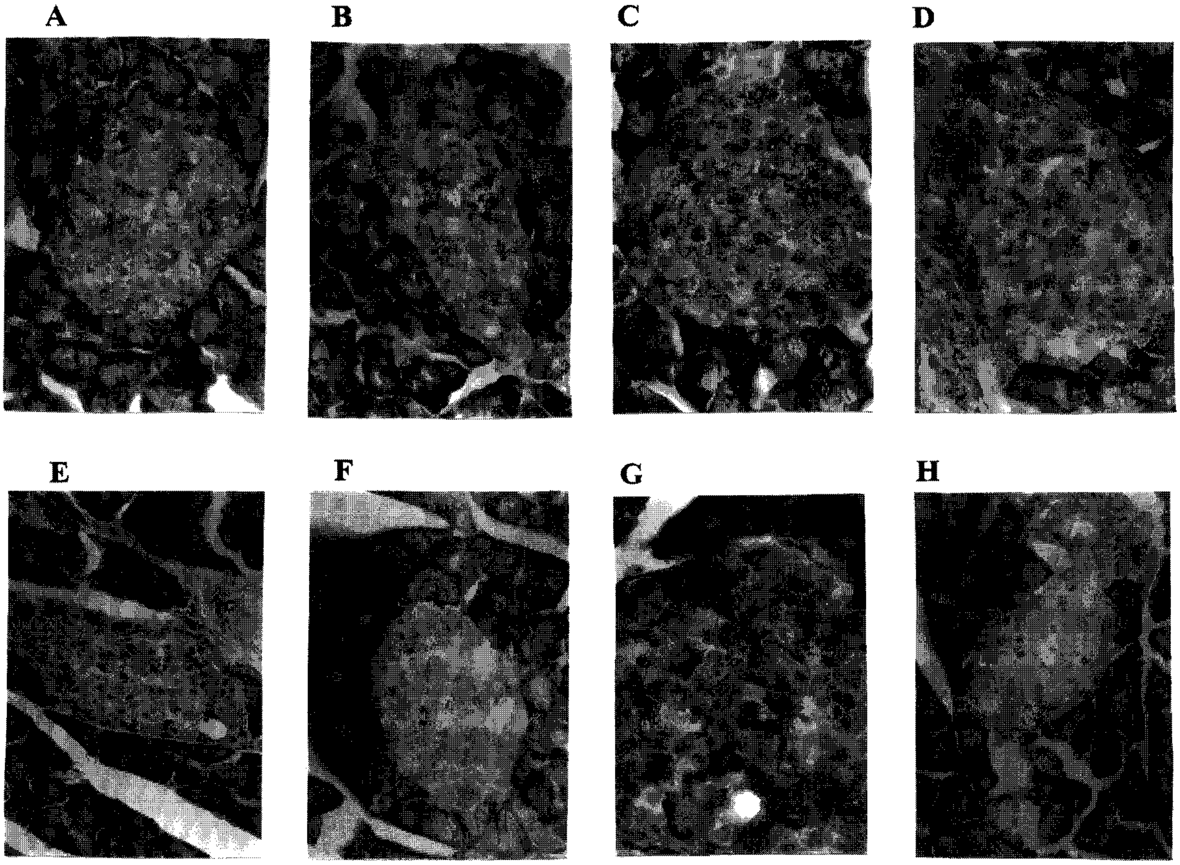

FIGURE 3 Effects of PTX treatment on the MLD-SZ-induced cellular changes in rat pancreas on day 10, as revealed by immunohistochemical staining (PAP). Upper panels, Control DA rats treated with MLD-SZ only. Note diffuse infiltration of mononuclear cells and the initial necrotic changes. Lower panels, DA rats treated with MLD-SZ + PTX. Note the absence of intrainsulitis with low degree of islet cell destruction. $CD4^+$ cells (W3/25 mAb), (A) mag. × 120 and (E) mag. × 120; $CD8^+$ cells (OX8 mAb), (B) mag. × 120 and (F) mag. × 120; $ED1^+$ blood-born macrophages (ED1 mAb), (C) mag. × 180 and (G) mag. × 180; MHC Class $II^+$ cells (OX6 mAb), (D) mag. × 180 and (H) mag. × 120

### DISCUSSION

In an attempt to elucidate the value of new drug regimens in human disorders, a number of animal models has been used with common molecular mechanisms that are influenced by the compund tested. For IDDM, widely accepted models similar to human disease are spontaneously developing diabetes in NOD mice and BB rats. By using these models it has been recently shown that PTX, and related compounds, rolipram (Liang et al., 1998) and theophylline (Rabinovitch and Sumoski, 1990) may favorably influence the course of the disease. However, multifactorial process resulting in clinically overt diabetes may differ, depending on the experimental model used for the study. In addition to genetic predisposition, various exogenic factors, like infective agents (Chung et al., 1997; Von Herrat et al., 1998.), dietary factors (Helgasson and Jonasson, 1981; Borch-Johnsen et al., 1984), or islet-specific toxins (Like and Rossini, 1976; Lenzen and Panten, 1988), may contribute to the development of the disease. Therefore, in order to examine immunomodulatory potential of PTX, we used the priming of islets with β-cell-specific toxin

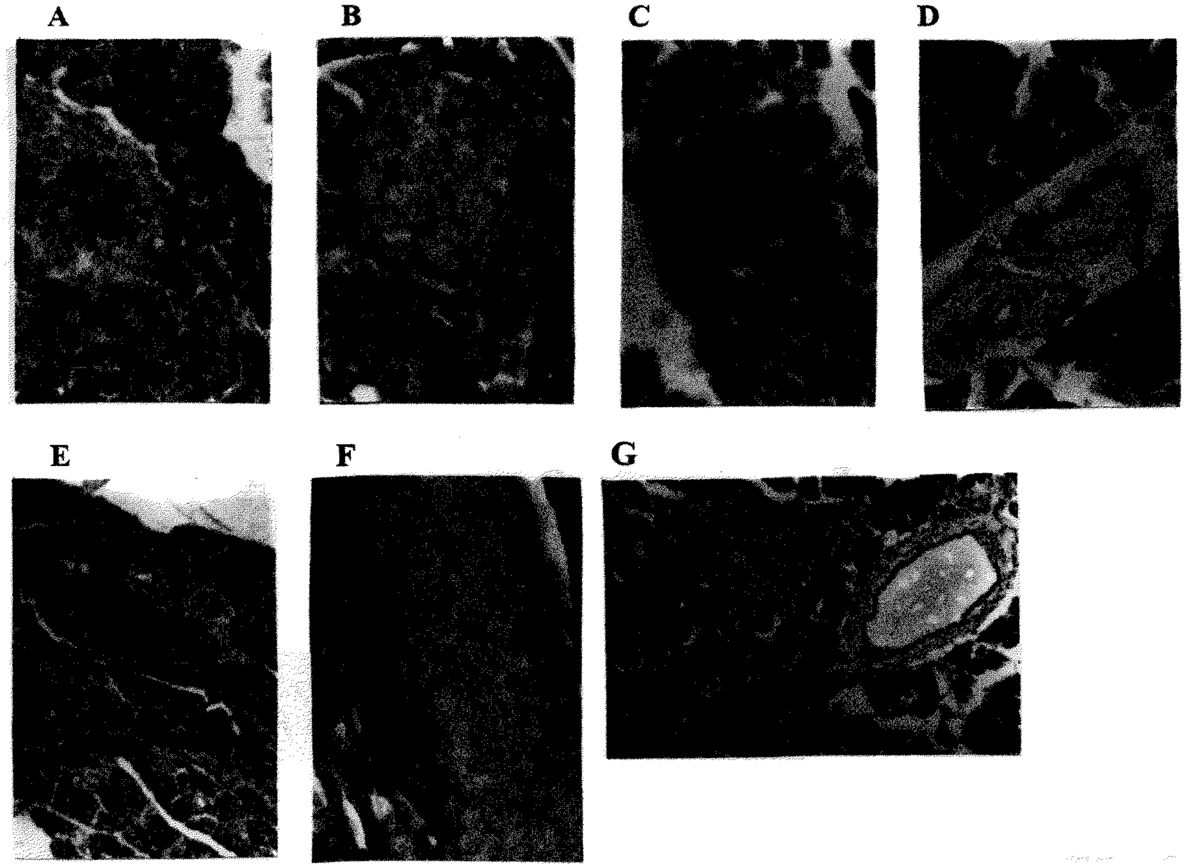

FIGURE 4 Effects of PTX treatment on the MLD-SZ-induced molecular changes in rat pancreas on day 10, as revealed by immunohistochemical staining of pancreata. AP staining to insulin, mag. × 120 (A) and mag. × 60 (E); IFN-$\gamma^+$ cells (DB1 mAb, PAP), mag. × 120 (B) and mag. × 120 (F); iNOS$^+$ cells (NO16 mAb, PAP), mag. × 120 (C), mag. × 180 (D) and mag. × 60 (G); Upper panels, Control DA rats treated with MLD-SZ only. Note paucity of insulin-containing cells restricted to the central area of the islet, diffuse pattern of IFN-γ containing cells, and homogenous pattern of iNOS-containing islet cells and endothelial cell layer. Lower panels, DA rats treated with MLD-SZ + PTX. Note homogenous pattern of insulin-containing cells throughout the entire islet, and the absence of IFN-containing and iNOS-containing cells

SZ, *in vivo* the treatment leading to an immune mediated inflammation and clinically overt diabetes. After relatively short course of treatment with PTX, there was a long-lasting protective effect both in susceptible CBA/H mice and DA rats. Although PTX prophylaxis could not completely suppress MLD-SZ-induced mononuclear cell infiltration of the islets, it drastically reduced its severity. It has been recently shown that PTX inhibits ICAM-1 expression in monocytes (Neuner et al., 1997) and the adhesion of T lymphocytes to ICAM-1 and VCAM-1 (Gonzales-Amaro et al., 1998). On the other hand, it has been shown in MLD-SZ-induced diabetic mice that adhesion of lymphocytes to islet endothelium depends on the expression of VCAM-1 and ICAM-1 in the pancreas (Ludwig et al., 1999). Therefore, it seems that beneficial effect of PTX on MLD-SZ-induced pathway of immune attack of β-cells may reside, at least in part, in its capacity to interfere with the recruitment of immune and inflammatory cells from circulation. Indeed, we have shown that PTX protects both mice and rats from development of destructive intrainsulitis.

MLD-SZ-induced autoimmune diabetes appears to be a T cell-dependent disease (Elliot et al., 1997). Since T cell reactivity is regulated by APCs, in animal models of IDDM it has been postulated that the functional state of APCs is responsible for the progression of Th1-dependent destructive insulitis (Rothe and Kolb, 1998). It appears that by stimulating Th cells (Cockfield et al., 1989), MLD-SZ caused up-regulation of IFN-γ and MHC Class II expression that are required to propagate the autoimmune process leading to IDDM. IFN-γ production can be reduced by treatment with PTX (Figure 4 and Liang et al., 1998). In addition, the spatial distribution of MHC Class $II^+$ cells in control MLD-SZ-induced animals expressed scattered pattern, while in PTX-treated animals Class $II^+$ cells were found only in peri-insulitis (Figure 3). Thus, according to our results, it seems that PTX may influence the disease by down-regulating APCs.

In MLD-SZ induced autoimmune diabetes several mechanisms have been implicated in the pathological processes leading to β-cell dysfunction and death, including proinflammatory cytokine mediated induction of iNOS expression and NO production (Lukić et al., 1991b; Lukić et al., 1998). Both exogenous NO derived from nonendocrine islet cells (macrophages or endothelial cells) and NO generated by the β-cells itself may contribute ,to tissue destruction (Corbett et al., 1992; Kaneto et al., 1995; Corbett and McDaniel, 1995). Since PTX has been found to be able to inhibit the release of inflammatory cytokines (Van-Furth et al., 1994; Rieneck et al., 1995), and favor Th2 response (Liblau et al., 1995), the drug may indirectly interfere with the induction of iNOS. It fits well with the finding that PTX reduces deleterious effects of TNF-α on human islets (Ariasdiaz et al., 1995). Recently we showed that PTX may have opposite effects on iNOS expression in different cell types: inhibitory in macrophages and enhancing in astrocytes (Trajković et al., 1997). Although the cellular sources of iNOS in our experimental model may be both endocrine and nonendocrine cells, in the present study we showed that local expression of iNOS by both intraislet cells and endothelial cells were reduced after *in vivo* treatment with PTX. From this, it can be concluded that another mechanism by which PTX could exert its effect is by reducing NO-mediated destruction of pancreatic β-cells Whatever the cellular source of NO is, interference with local NO production by PTX could be relevant for autoimmune process affecting the pancreas. However, the precise mechanism of action on NO synthesis at the level of NO-producing cells in the pancreas is at present not known, but warrants further study.

### MATERIALS AND METHODS

### Animals

Genetically susceptible inbred male Dark Agouti (DA) rats, and CBA/H mice were obtained from our own breeding colony (Institute for Biological Research, Belgrade, Yugoslavia), determined to be free from common pathogens. Animals were used when 10 to 16 weeks old, and kept in groups of 5 to 6 per cage.

### Induction of Diabetes and Drug Treatment

Diabetes was induced in two rodent species with multiple subtoxic doses of streptozotocin (SZ, S-0130, Sigma, St Louis, MO), (20 mg/kg b. w. /day in rats, and 40 mg/kg b. w. /day in mice), given i.p. for 5 consecutive days. Pentoxyfilline (PTX, Panfarma, Belgrade, Yugoslavia) was given i.m. at a dose of 200 mg/kg/day, from day 0 through day 9 in relation to the induction of diabetes. Control, nontreated animals received injections of PBS. Plasma glucose was determined by a glucose-oxidase method using a glucometer (Glucotronic C; Macherey-Nagel, Duren, Germany) once a week for up to 8 weeks. Clinical diabetes was defined by hyperglycemia in nonfasted animals (blood glucose > 11 mmol/l).

### Histological and Immunohistochemical Analyses of Pancreas

For histology, pancreata were prepared by embedding in paraffin after fixing in formalin. To assess the incidence of inflammatory changes, degree of islet cell destruction and changes of connective tissue, histologic sections (7 μm in thickness) were stained with hematoxylin and eosin. Histological analysis was performed in a blind fashion by two observers. The degree of inflammatory changes was graded according to the following arbitrary scale: 0 = intact islet with no cellular infiltrates, 1 = few infiltrated intraislet cells, but intact islet architecture, and 2 = heavy mononuclear infiltration with or without loss of islet architecture. At least 5 animals per each experimental group, and minimum 20 islets per animal were scored individually.

Immunohistochemical analysis was performed from a snap-frozen sections, using indirect immunoperoxidase staining as described previously (Stošić-Grujičić et al., 1999). Staining of rat tissue was performed using the following mouse anti-rat mAbs: anti-CD4 (W3/25, Serotec, Oxford, England), anti-CD8 (OX 8, Serotec), anti-IFN-γ (DB1, Holland Biotechnology, Leiden, The Netherlands), anti-MHC Class II (OX-6, Holland Biotechnology), and anti-monocytes and macrophages (ED1, kindly provided by Dr. C. D. Dijskstra, Medical Faculty, Free University, Amsterdam, The Netherlands). Rabbit antiserum raised against a synthetic peptide corresponding to the C-terminus of a long form of mouse macrophage iNOS (NO16; kindly provided by Dr. C. Nathan, Cornell University Medical College, NY), cross-reactive with rat iNOS, was used for tissues of both rodent species. Primary mAbs for mouse tissue were as follows: rat anti-mouse CD4 (GK1.5) and CD8 (YTS 169), both from Serotec, Oxford, and anti-mouse interferon-gamma (IFN-γ), (F3, Holland Biotechnolgy). Staining to insulin was performed with guinea pig anti-swine insulin primary antibody (N 1542, Dako, Hamburg, Germany) using DAKO LSAB Kit (K 0676, Dako) with alkaline phosphatase (AP)-conjugated rabbit/mouse secondary antibody.

### Statistical Analysis

Data were expressed as means ± s.e.m. Student's *t* test was used for evaluation statistical significance of differences between groups. P-values were considered significant at $P < 0.05$.

### *Acknowledgements*

This work was supported by grants from Ministry of Science and Technology, Republic of Serbia, Yugoslavia. M. L. Lukić's research was supported by FMHS grant of UAE. Staining to insulin was kindly performed by Dr. V. Bumbaširević, School of Medicine, Belgrade University, Belgrade, Yugoslavia.

### *References*

Ariasdiaz J., Vara E., Garcia C., Torresmelero J., Rodrigez J. M., and Balibrea J. L. (1995). Pentoxifylline partially reverts the effect of tumor necrosis factor on human islets. Transplant. Proc. **26**:698–700.

Borch-Johnsen K., Mandrup-Poulsen T., Zachau-Christiansen B., Joner G., Bhristy M., Kastrup K., and Nerup J. (1984). Relation between breast-feeding and incidence rates of insulin-dependent diabetes mellitus. Lancet **1**:1083–1086.

Chung Y.H., Jun H.-S., Kang Y., Hirasawa K., Lee B.-R., Rooijen N. V., and Yoon J.-W. (1997). Role of macrophages and macrophage-derived cytokines in the pathogenesis of Kilham rat virus – induced autoimmune diabetes in diabetes – resistant BioBreeding rats. J. Immunol. **159**:466–471.

Cockfield S. M., Ramassar V., Urmson J., and Halloran P. F. (1989). Multiple low dose streptozotocin induces systemic MHC expression in mice by triggering T cells to release IFN-γ. J. Immunol. **142**:1120–1128.

Constantinescu S.C., Hillard B., Lavi E., Ventura E., Venkatesh V., and Rostami A. (1996). Suppression of experimental autoimmune neuritis by phosphodiesterase inhibitor pentoxifylline. J. Neurol. Sci. **143**:14–18.

Corbett J. A., and McDaniel M. L. (1995). Intraislet release of interleukin 1 inhibits β cell expression of inducible nitric oxide synthase. J. Exp. Med. **181**:559–565.

Corbett J. A., Wang J. L., Sweetland M. A., Lancaster J. R., and McDaniel M. L. (1992). IL-1β induces the formation of nitric oxide by β-cells purified from rodent islets of Langerhans. J. Clin. Invest. **90**:2384–2391.

Elliot J. I., Dewchand H., and Altmann D. M. (1997). Streptozotocin induced diabetes in mice lacking αβ T-cells. Clin. Exp. Immunol. **109**:116–120.

Gonzales-Amaro R., Portales-Perez D., Baranda L., Redondo J. M., Martinez-Martinez S., Yanez-Mo M., Garcia-Vicuna R., Cabanas C., and Sanches-Madrid F. (1998). Pentoxifylline inhibits adhesion and activation of human T lymphocytes. J. Immunol. **161**:65–72.

Helgasson T., and Jonasson M. R. (1981). Evidence for a food additive as cause for ketosis-prone diabetes. Lancet **II**:716–720.

Kaneto H., Fujii J., Seo H. G., Suzuki K., Matsuoka T.-A., Nakamura M., Tatsumi H., Yamasaki Y., Kamada T., and Taniguchi N. (1995). Apoptotic cell death triggered by nitric oxide in pancreatic β-cells. Diabetes **44**:733–738.

Kolb H., Kroncke K.-D. (1993). IDDM: lessons from the low-dose streptozotocin model in mice. Diabetes Rev. **1**:116–126.

Lenzen S., and Panten U. (1988). Alloxan: history and mechanisms of action. Diabetologia **31**:337–342.

Liang L., Beshay E., Prud'homme G. J. (1998). The phosphodiesterase inhibitors Pentoxifylline and Rolipram prevent diabetes in NOD mice. Diabetes **47**:570–575.

Liblau R. S., Singer S. M., and McDevitt H. O. (1995). Th1 and Th2 $CD4^+$ T cells in the pathogenesis of organ-specific autoimmune diseases. Immunol. Today **16**:34–38.

Like A.A., Rossini A.A. (1976). Streptozotocin-induced pancreatic insulitis: a new model of diabetes mellitus. Science **193**:415–417.

Ludwig R., Kretschmer M., Caspar G., Bojunga J., Oldenburg A., Schumm-Draeger P., Stegmüller M., Von Minckwitz G., Usadel K.-H., and Kusterer K. (1999). *In vivo* microscopy of murine islets of Langerhans: increased adhesion of transferred lymphocytes to islets depends on macrophage-derived cytokines in a model of organ-specific insulitis. Immunology **98**:111–115.

Lukić M.L., Al-Sharif R., Mostarica M., Bahr G., and Behbehani K. (1991a). Immunological basis of the strain differences in susceptibility to low-dose streptozotocin-induced diabetes in rats. In Lymphatic Tissues and In Vivo Immune Responses, Imhof, et al., Eds. (New York: Marcel Dekker), pp. 643–647.

Lukić M. L., Stošić-Grujičić S., Ostojić N., Chan W. L., and Liew F. Y. (1991b). Inhibition of nitric oxide generation affects the induction of diabetes by streptozotocin in mice. Biochem. Biophys. Res. Commun. **178**:913–920.

Lukić M.L., Stošić-Grujičić S., and Shahin A. (1998). Effector mechanisms in Low Dose streptozotocin-induced diabetes. Dev. Immunol. **6**:119–128.

Nataf S., Louboutin J. P., Chabannes D., Feve J. R., and Muller J. Y. (1993). Pentoxifylline inhibits experimental allergic encephalomyelitis. Acta Neurol. Scand. **88**:97–99.

Neuner P., Klosner G., Pourmojib M., Knobler R., and Schwarz T. (1997). Pentoxifylline *in vivo* and *in vitro* down-regulates the expression of the intercellular adhesion molecule-1 in monocytes. Immunology **90**:435–439.

Rabinovitch A., and Sumoski W. L. (1990). Theophylline protects against diabetes in BB rats and potentiates cyclosporin protection. Diabetologia **33**:506–508.

Rieneck K., Diamant M., Haahr P. M., Schonharting M., and Bendtzen K. (1995). *In vitro* immunomodulatory effects of pentoxifylline. Immunol Letters **37**:131–138.

Rothe H., and Kolb H. (1998). The APC1 concept of type I diabetes. Autoimmunity **27**:179–184.

Rott O., Cash E., Fleischer B. (1993). Phosphodiesterase inhibitor pentoxifylline, a selective suppressor of T helper type 1- but not type 2-associated lymphokine production, prevents induction of experimental autoimmune encephalomyelitis in Lewis rats. Eur. J. Immunol. **23**:1745–1751.

Saez-Lorens X., Ramilo O., Mustafa M. M., Mertsola J., De Alba C., Hansen E., and McCracken G. H. (1990). Pentoxifylline modulates meningeal inflammation in experimental bacterial meningitis. Antimicrob. Agent Chemother. **34**:837–843.

Stošić-Grujičić S., Dimitrijević M., and Bartlett R. R. (1999). Leflunomide protects mice from multiple low dose streptozotocin (MLD-SZ)-induced insulitis and diabetes. Clin. Exp. Immunol. **117**:44–50.

Trajković V., Badovinac V., Popadić D., Hadžić O., and Stojković M.M. (1997). Cell-specific effects of pentoxifylline on nitric oxide production and inducible nitric oxide synthase mRNA expression. Immunology **92**:402–406.

Van-Furth A. M., Steenwijk T. M., Langermans J. A., and Van-Furth R. (1994). *In vitro* effect of dexamethasone, pentoxifylline, and anti-endotoxin monoclonal antibody on the release of proinflammatory mediators by human leukocytes stimulated with haemophilus influenzae type B. Pediatr. Res. **35**:725–728.

Von Herrat M. G., Holz A., Homman D., and Oldstone M. B. (1998). Role of viruses in type I diabetes. Semin. Immunol. **10**:87–100.

